# Potential Inhibitory Effect of the Peptide Melittin Purified from *Apis mellifera* Venom on CTX-M-Type Extended-Spectrum β-Lactamases of *Escherichia coli*

**DOI:** 10.3390/antibiotics14040403

**Published:** 2025-04-14

**Authors:** Sheril Ramos-Alcántara, María Alejandra Cornejo Napan, Giovanni Lopez Campana, Jesus Tamariz

**Affiliations:** 1 Laboratorio de Resistencia Antibiótica y Fagoterapia, Facultad de Medicina, Universidad Peruana Cayetano Heredia, Lima 15102, Peru; maria.cornejo.n@upch.pe; 2 Instituto de Medicina Tropical Alexander von Humboldt, Universidad Peruana Cayetano Heredia, Lima, 15102, Peru; mariano.lopez.c@upch.pe

**Keywords:** antimicrobial peptides, Melittin, ESBL, enzymatic activity, Apitoxin

## Abstract

**Background.** Extended-spectrum β-lactamases (ESBLs) hydrolyze nearly all β-lactam antibiotics, affecting one of the most important groups of antimicrobials used in Gram-negative infections. Among them, CTX-M is the most widespread type of ESBL. This study aimed to evaluate the hydrolytic activity of CTX-M-type ESBLs following exposure to the antimicrobial peptide Melittin. **Methods.** Melittin was purified from *Apis mellifera* venom through ultrafiltration and characterized by SDS-PAGE. The minimum inhibitory concentration (MIC) of Melittin against ESBL-producing *E. coli* was determined by the broth microdilution method. The inhibition of ESBL’s hydrolytic activity following exposure to sub-MIC doses of Melittin was quantified using a kinetic assay based on hydrolyzed nitrocefin. Additionally, the effect of Melittin on the expression of the *bla*_CTX-M_ gene was evaluated via RT-PCR. **Results.** The peptide fraction of Apitoxin smaller than 10 kDa exhibited a protein band corresponding to Melittin, devoid of higher molecular weight proteins. The MIC of Melittin ranged from 50 to 80 µg/mL. Exposure to Melittin at sub-MIC doses significantly inhibited ESBL hydrolytic activity, reducing it by up to 67%. However, the transcription of the *bla*_CTX-M_ gene in the presence of Melittin revealed no significant changes. **Conclusions**. Melittin is able to inhibit ESBL’s hydrolytic activity but not *bla*_CTX-M_ transcription possibly indicating an effect at the translational or post-translational level.

## 1. Introduction

Extended-spectrum β-lactamases (ESBLs) represent the most widespread mechanism of antibiotic resistance globally [1]. ESBL enzymes hydrolyze the β-lactam ring, rendering the majority of β-lactam antibiotics, including penicillins, monobactams, and extended-spectrum cephalosporins [2]. Over the past two decades, the CTX-M family has emerged as the dominant type of ESBL in clinical *Escherichia coli* isolates, driven by the rapid dissemination of the *bla*_CTX-M_ gene [3,4]. In May of this year, the World Health Organization ratified the critical public health threat posed by ESBL-producing *Enterobacteriaceae*, underscoring their global impact on disease burden, transmissibility, treatment challenges, and limited preventive options. This prioritization calls for intensified research and the development of new therapeutic alternatives [5].

Currently, the use of β-lactamase inhibitors remains the treatment of choice, for neutralizing already formed or active ESBLs enzymes. However, these inhibitors face limitations such as enzyme-specific affinity, as well as discrepancies between in vitro susceptibility tests and clinical efficacy [6]. An alternative and less explored strategy involves developing compounds that interfere with the synthesis or hydrolytic activity of ESBL enzymes. In this context, antimicrobial peptides have demonstrated inhibitory effects on bacterial metabolic pathways by interfering with cell wall synthesis, nucleic acid synthesis, protein synthesis, enzymatic activity, and the production of exoenzymes, hemolysins, and toxins [7,8]. Antimicrobial peptides (AMPs) are small bioactive proteins generally composed of 10–50 amino acids with a molecular weight of less than 10 KDa. Most AMPs are positively charged, derived primarily from lysine and arginine (rarely histidine) in the specific cationic domain [9].

Natural products contain diverse molecules of great structural complexity, becoming a significant source of novel therapeutic agents [10]. Many successful antimicrobials developed by the pharmaceutical industry have been isolated from plants, microbes, and marine organisms [11]. The venom of the European honeybee (*Apis mellifera*), commonly referred to as Apitoxin, is a complex mixture of biologically active components, including proteins, peptides, and other molecules in smaller concentrations [12]. Among these, Melittin is the principal peptide component, accounting for 40–60% of the dry weight of Apitoxin [13]. Melittin is a potent AMP composed of 26 amino acid residues with a molecular weight of 2.8 kDa, and it exhibits antibacterial activity against a wide range of microorganisms [14]. The cationic nature of Melittin allows for strong electrostatic binding to negatively charged bacterial structures [15]. Furthermore, the C-terminal structural portion of Melittin may act as an anchor to lipopolysaccharides (LPS) in the outer membrane of Gram-negative bacteria, facilitating peptide translocation to other intracellular targets [16].

At sub-inhibitory doses, Melittin has been shown to interfere with nucleic acid synthesis in *Xanthomonas oryzae*, a rice pathogen [17], and to induct an apoptotic pathway in *Candida albicans* [18]. Based on this evidence, the objective of this study was to evaluate the hydrolytic activity of CTX-M-type ESBLs from *E. coli* following in vitro exposure to sub-inhibitory concentrations of purified Melittin. This investigation was further complemented by gene expression analyses of the *bla*_CTX-M_ gene to assess the effects of sub-inhibitory doses on β-lactamase transcription.

## 2. Results

### 2.1. Protein Analysis of Melittin

**Figure 1:**
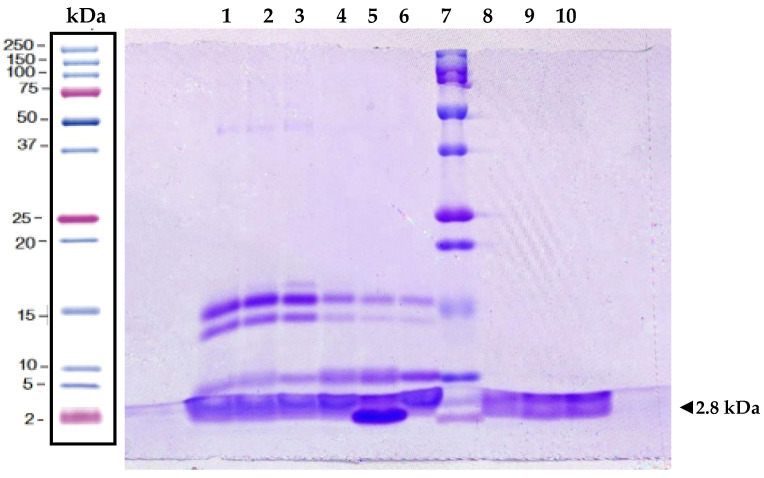
Melittin and bee venom separation by SDS-PAGE at 19%. Lanes 1–3: Purified Apitoxin from *Apis mellifera*, including high molecular weight proteins such as hyaluronidases and phospholipases. Lanes 4–6: Api < 30 kDa fraction containing phospholipases and Melittin. Lane 7: Precision Plus Protein™ Dual Xtra Standards Marker (Bio-Rad, Carlsbad, CA, USA). Lane 8: Commercial Sigma Melittin, isolated from *Apis mellifera* venom and lyophilized (>85% HPLC). Lane 9–10: Api < 10 kDa fraction with isolated Melittin.

Pure Melittin was successfully extracted from Apitoxin (Figure 1). The presence of pure Apitoxin was confirmed by the detection of protein bands with molecular weights corresponding to the most abundant proteins in its composition: Hyaluronidases (45 kDa), Phospholipase A2 isoforms (14–16 kDa), and Melittin (2.8 kDa). In the Apitoxin fraction smaller than 10 kDa (Api < 10 kDa), Melittin was identified with a purity greater than 99%, as determined by densitometric analysis using ImageJ version 1.54g (National Institutes of Health, Bethesda, MD, USA, https://imagej.net/ij/, accessed on 14 January 2025). The molecular weight of this isolated Melittin matched that of commercial Melittin (>85% HPLC purity, Merck, St. Louis, MO, USA), thus confirming its identity.

Melittin was the predominant protein, accounting for 61.52% of the total protein content in pure Apitoxin and 89.4% in the Apitoxin fraction smaller than 30 kDa (Api < 30 kDa). Additionally, a possible tetramerized form of Melittin (~11 kDa) was observed in pure Apitoxin and Api < 30 kDa fraction. The total protein concentration in the Api < 10 kDa fraction was 321 µg/mL.

### 2.2. Antimicrobial Activity of Melittin

The antimicrobial activity of Melittin was evaluated against five ESBL-producing *E. coli* strains. Table 1 summarizes the Minimal inhibitory concentration (MIC) and sub-MIC values for each strain in the presence of Melittin. The average MIC for the tested strains was 62 µg/mL. The highest MIC value, 90 µg/mL, was observed for strain *K. pneumoniae* ATCC 700603, while the lowest MIC, 30 µg/mL, was recorded for strain *E. coli* ATCC 25922.

**Figure 2:**
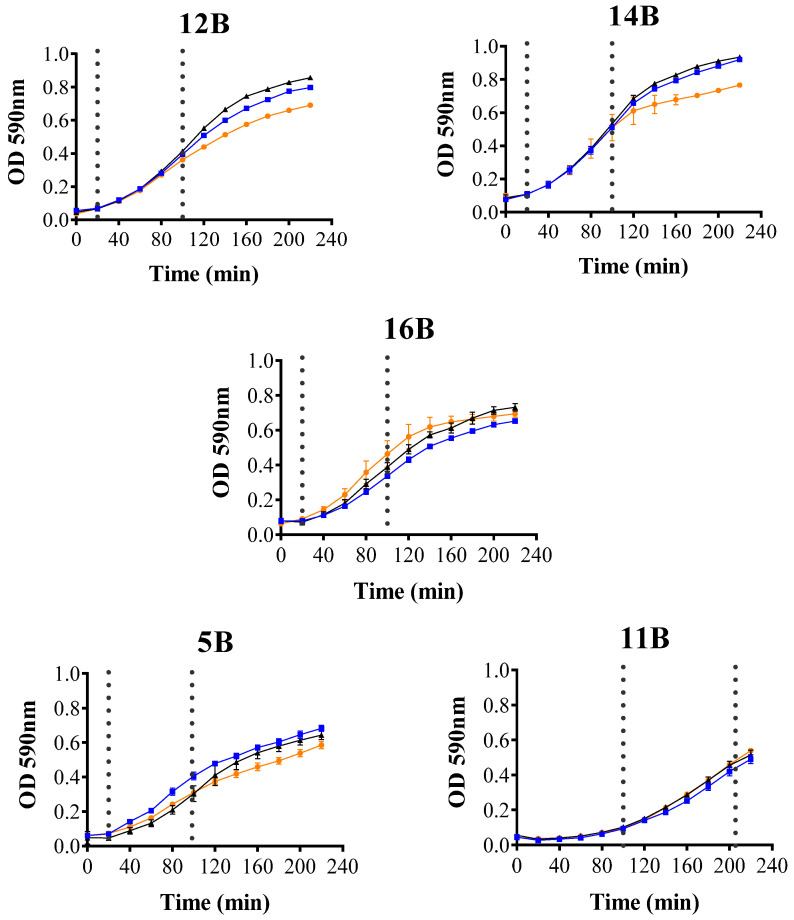
Growth curves of CTX-M-type ESBL-producing *E. coli* strains exposed to sub-inhibitory doses of Melittin. Doses: 0 (●), 1/2 MIC (▲), and 1/4 MIC (■) µg/mL of Melittin. The culture was performed in triplicate. Error bars represent the variability across the three replicates. The dashed lines indicate the start and end of the average logarithmic phase.

The effect of Melittin on the exponential growth phase of ESBL-producing *E. coli* strains was also assessed. As illustrated in Figure 2, growth curves in the presence and absence of Melittin were similar across all five strains (*p* > 0.05). These results indicate that Melittin does not inhibit the early growth phases of ESBL-producing *E. coli.*

### 2.3. Effect of Melittin on Hydrolytic Activity

Sub-inhibitory concentrations of Melittin (1/2 MIC and 1/4 MIC) were added to exponential phase cultures of ESBL-producing *E. coli* strains. The standard curve for hydrolyzed nitrocefin exhibited a strong correlation coefficient (r^2^ = 0.9995). A dose-dependent reduction in ESBL enzymatic activity was observed (Table 2).

At a concentration of 1/2 MIC, Melittin significantly reduced ESBL enzymatic activity (*p* < 0.02), with an average inhibition of 40%. The highest Melittin concentration tested, 40 µg/mL (equivalent to 14 µM), achieved a 67% inhibition of CTX-M-type ESBL hydrolytic activity. In contrast, the 1/4 MIC concentration showed a less pronounced inhibitory effect, reducing enzymatic activity in only two of the five strains (14B and 16B).

Baseline enzymatic activity in *E. coli* ESBL strains without Melittin exposure (controls) ranged from 294 mU/mg to 4589 mU/mg of protein. Additionally, the control strain for ESBL enzyme production, *K. pneumoniae* ATCC 700603, exhibited an enzymatic activity of 1105 mU/mg of protein.

### 2.4. Effect of Melittin on bla_CTX-M_ Expression

Melting curve analysis of RT-qPCR reactions showed a unique peak of the amplified products. However, strain 12B was excluded from further analysis due to the presence of an additional peak.

**Figure 3:**
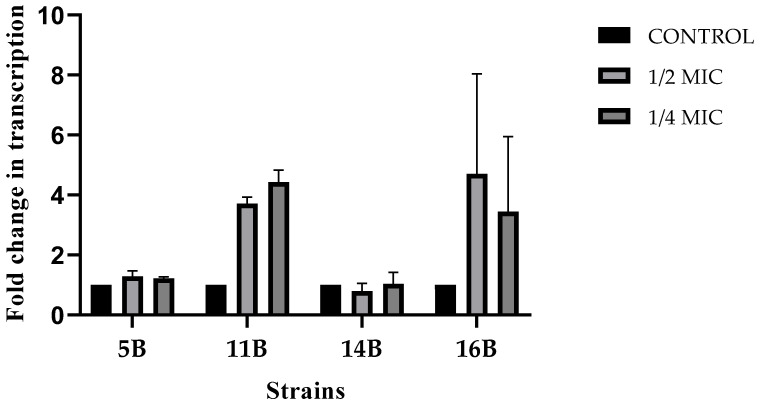
Fold change normalized to the reference gene 16S rRNA in *bla*_CTX-M_ transcription after supplementation with sub-inhibitory concentrations of Melittin, stratified by strain.

Gene expression levels of the *bla*_CTX-M_ and the 16S rRNA were evaluated after exposure to sub-inhibitory concentrations of Melittin (Figure 3). Strains exposed to 1/2 MIC and 1/4 MIC of Melittin exhibited a non-significant increase in *bla*_CTX-M_ gene expression compared to the control group (*p* > 0.27).

## 3. Discussion

Currently, three β-lactamase inhibitors containing a β-lactam ring are available for treating CTX-M-type ESBL; however, resistance to these β-lactam/inhibitor combinations has been reported [19,20]. This highlights the urgent need to develop novel therapeutic strategies targeting alternative sites to combat ESBL resistance and preserve the efficacy of β-lactam antibiotics. Naturally derived antimicrobial peptides, recognized as bioactive molecules with significant pharmacological potential, offer promising avenues for antibiotic-resistant bacterial infections [9,21].

This study aimed to evaluate the ability of the antimicrobial peptide Melittin, derived from *Apis mellifera* venom, to interfere with the enzymatic activity of CTX-M-type ESBLs in *E. coli*. The results demonstrated that sub-inhibitory concentrations of Melittin (1/2 MIC and 1/4 MIC) reduced ESBL hydrolytic activity in a dose-dependent manner. At 1/2 MIC, Melittin achieved maximum and minimum enzymatic activity inhibition of 67.2% and 24%, respectively. This inhibition would rendered ESBL enzymes ineffective to hydrolyzing β-lactams, allowing them to reach its specific target and consequently exert their bactericidal action. These findings are consistent with previous studies demonstrating Melittin’s synergistic effects with β-lactam antibiotics against multidrug-resistant *Acinetobacter baumannii* and *Pseudomonas aeruginosa* strains [22,23,24,25].

Recent studies have also proposed that Melittin may bind to and inhibit carbapenemases, a class of β-lactamases, thereby explaining its synergy with imipenem against carbapenem-resistant *A. baumannii* [26]. Similarly, other compounds with different chemical structures have demonstrated interference with β-lactamase hydrolytic activity. For example, glycerol monolaurate disrupts intracellular signaling in *Staphylococcus aureus bla*_Z_ cultures, inhibiting β-lactamase induction and significantly reducing enzymatic activity [27].

To further investigate Melittin’s effect on ESBL’s enzymatic activity, the regulation of *bla*_CTX-M_ expression was evaluated under sub-inhibitory concentrations (1/2 MIC and 1/4 MIC). Although no changes were observed in *bla*_CTX-M_ expression comparing treated and untreated strains, the possibility of post-translational regulatory mechanisms cannot be excluded. The production of β-lactamase enzymes in the cytoplasm involves complex biochemical pathways, often assisted by chaperones, where enzymatic activity depends on correct protein folding [28,29]. In methicillin-resistant *S. aureus* strains, Melittin has been shown to inhibit the expression of proteins involved in protein synthesis, such as elongation factor G (EF-G) and the chaperone DnaK, which are essential for protein assembly and preventing aggregation [30]. Furthermore, ESBLs in Enterobacteriaceae are extracellular enzymes requiring translocation to the periplasm to achieve their active soluble form [28]. These processes, which depend on ATP hydrolysis, are critical for ESBL activity. At a concentration of 9.03 µM, Melittin has been reported to inhibit 50% of the activity of *E. coli* F0F1 ATP synthase, an enzyme crucial for ATP-dependent processes [31]. Similarly, the secretion of periplasmic proteins, including pre-β-lactamases in *E. coli*, was inhibited by Cerulenin, resulting in a >70% reduction in β-lactamase activity [32]. Further molecular studies are needed to elucidate the precise mechanisms by which Melittin affects these essential bacterial enzymes.

Variability in CTX-M-type ESBL enzymatic activity was observed within the control group not exposed to Melittin, likely due to the presence of different CTX-M variants. For example, previous studies have reported enzymatic activity values of 2700 and 724 nmoles/min/mg protein for the CTX-M-10 and CTX-M-1 variants, respectively [33,34]. Additionally, enzymatic activity may vary depending on factors such as the location of the *bla* gene, the presence of β-lactamase inducers like cefotaxime, and the bacterial growth phase [35]. In this study, ESBL activity was assessed during the logarithmic growth phase, which is associated with high CTX-M production in the absence of inducers. While antimicrobial peptides like Melittin can delay the lag phase of bacterial growth [36], sub-inhibitory concentrations of Melittin did not affect the growth of ESBL-producing *E. coli* during early or mid-logarithmic phases, suggesting that its inhibitory effect on ESBL enzymes is specific.

Melittin was purified from *Apis mellifera* venom using ultrafiltration, which proved effective for concentrating peptides with a purity comparable to that achieved by High-Performance Liquid Chromatography (HPLC) [37]. Electrophoretic characterization showed that Melittin was concentrated in the <10 kDa fraction, indicating successful removal of high molecular weight proteins such as hyaluronidases and phospholipases A2. Many bioactive peptides have been purified using similar membrane-based techniques. For instance, Beaubier et al. successfully purified the antimicrobial peptide neokyotorphin (NKT) using regenerated cellulose membranes with a 1–3 kDa cut-off [38]. Although the <10 kDa fraction may contain other minor peptides like apamin and mast cell degranulating peptides, their biological effects are negligible compared to Melittin [39]. Overall, ultrafiltration is a cost-efficient and effective method for purifying bioactive peptides of low molecular weight.

The antimicrobial activity of peptides can be influenced by transition metals. For example, in CA-MHB supplemented with divalent cations (Ca^2+^ and Mg^2+^), the MIC of Melittin against *E. coli* clinical strains was 30 µg/mL [40]. Conversely, in cation-free MHB, the MIC for ESBL-producing *E. coli* was lower (MIC_90_ = 10 µg/mL) [41]. Consistent with these observations, this study determined a MIC_90_ of 60 µg/mL for ESBL-producing *E. coli* in CA-MHB, corroborating earlier findings that Melittin’s antimicrobial activity is reduced in the presence of divalent cations [42].

It is important to highlight that the evaluated *E. coli* ESBL strains exhibited varying behaviors in terms of bacterial growth, MIC of Melittin, and the degree of reduction in the hydrolytic activity of the ESBL enzyme. These differences may be attributed to the specific characteristics of the studied strains or variations in the allelic forms of the *bla*_CTX-M_ gene, which were not assessed and represent a limitation of this study. Another limitation of this study is the absence of cytotoxicity tests for Melittin, which are important and should be conducted in future studies as this line of research progresses.

The results obtained do not conclusively establish Melittin as a viable candidate for the development of antimicrobial drugs. However, they provide compelling evidence of its inhibitory effect on the hydrolytic activity of CTX-M-type ESBL. This observation warrants further investigation to explore its potential as a novel target for action against β-lactamases.

In conclusion, exposure to Melittin reduces the hydrolytic activity of CTX-M-type ESBLs in *E. coli*. Although transcript expression remained unchanged, the observed inhibition may result from translational or post-translational modifications.

## 4. Methods

### 4.1. Bacterial Strains

Five *E. coli* strains producing ESBL were selected from a previous study [43]. These strains lacked enzymatic resistance mechanisms such as carbapenemases and AmpC-type β-lactamases. All strains were reactivated in Luria broth (Merck, St. Louis, MO, USA) and incubated at 37 °C with shaking at 150 rpm (Biobase, Jinan, China). The functional activity of ESBL enzymes was confirmed using the Bauer–Kirby disk diffusion method [44]. PCR analysis confirmed that these strains harbored the *bla*_CTX-M_ gene but lacked *bla*_TEM_ and *bla*_SHV_. Additionally, the ESBL-producing strain *Klebsiella pneumoniae* ATCC 700603 (SHV-18) and a non-ESBL-producing strain, *E. coli* ATCC 25922, were included.

### 4.2. Purification and Characterization of Melittin

Crystals of Apitoxin were collected from healthy apiaries in northern Peru during a previous study and stored at −20 °C until use [45]. Apitoxin was purified following the methodology described by Cuya [46]. Briefly, Apitoxin crystals were dissolved in sterile deionized water at a 1% concentration. The suspension was filtered through a 0.22 µm membrane to obtain pure Apitoxin, which was then processed using ultrafiltration membranes (Amicon^®^ Merck, Darmstadt, Germany) with a 30 kDa molecular weight cut-off. After centrifugation at 4000× *g* for 30 min, the resulting Apitoxin fraction smaller than 30 kDa (Api < 30 kDa) was further ultrafiltered using a 10 kDa cut-off membrane. The resulting new eluate represented the pure Apitoxin peptide fraction lesser than 10 kDa (Api < 10 kDa), which contained Melittin (MW = 2.846 kDa). Protein concentrations of pure Apitoxin and its fractions were determined using fluorometry (Qubit™ Protein Assay Kit, Invitrogen, Thermo Fisher Scientific, Eugene, OR, USA). The purity of Apitoxin and its fractions was verified by polyacrylamide gel electrophoresis (19% SDS-PAGE). A commercially available Melittin with >85% purity (HPLC, Sigma-Aldrich, Saint Louis, MO, USA) was used as a reference standard.

### 4.3. Determination of the Minimum Inhibitory Concentration (MIC) of Melittin

The antibacterial activity of Melittin was determined by calculating its MIC using the broth microdilution method, following CLSI guidelines [47] with modifications. Melittin was diluted in cation-adjusted Mueller–Hinton broth (CA-MHB, Liofilchem, Roseto degli Abruzzi, Italy) in a 96-well polystyrene microplate at the following concentrations: 100, 90, 80, 70, 60, 50, 40, 30, 20, and 10 µg/mL, with a final volume of 100 µL per well. The bacterial inoculum was prepared from isolated colonies of *E. coli* (ESBL-producing and ATCC 25922) and *K. pneumoniae* cultures at a concentration equivalent to 0.5 McFarland (1.5 × 10⁸ CFU/mL). This suspension was diluted 1:20 in sterile saline solution (0.9% NaCl), and 10 µL volumes were added to the wells containing Melittin (final bacterial concentration: 5 × 10⁵ CFU/mL). Plates were incubated at 37 °C for 18 h. The MIC was defined as the lowest concentration of the antimicrobial agent at which no visible bacterial growth occurred. The experiment was performed in triplicate.

### 4.4. Growth Curve Assay

The capacity of Melittin to alter bacterial growth from the initial to the late logarithmic phase was evaluated following the methodology of Dolzani L. et al. [48], with modifications. Melittin was diluted in CA-MHB at sub-MIC concentrations (1/2 MIC, 1/4 MIC) specific to the MIC of each bacterial strain, and 200 µL was transferred to a 96-well microplate. Overnight bacterial cultures were grown in 10 mL of CA-MHB at 37 °C without shaking. Subsequently, 10 mL of fresh CA-MHB was added, and the cultures were incubated with shaking at 240 rpm for 1 h (Biobase, Jinan, China). The early-log phase cultures obtained served as the bacterial inoculum, and 10 µL of this inoculum was added to each well containing Melittin (final bacterial concentration: 10^7^ CFU/mL). Bacterial growth was monitored by measuring optical density (OD) at 590 nm every 20 min for 4 h at 37 °C with intermittent shaking using a microplate reader (Tecan Trading AG, Männedorf, Switzerland).

### 4.5. Measurement of Hydrolytic Activity

The hydrolytic activity of β-lactamases in *E. coli* ESBL-producing strains and *K. pneumoniae* ATCC 700603 was evaluated following the protocol of Brown-Skrovot et al. [27], with modifications. Cultures were initially grown overnight in 10 mL of CA-MHB at 37 °C without shaking. Afterward, 10 mL of fresh CA-MHB was added, and cultures were incubated with shaking at 240 rpm for 1 h. Early-log phase cultures were diluted 1:20 in CA-MHB containing Melittin to prepare final subcultures of 50 mL (final bacterial concentration: 10^7^ CFU/mL). Melittin was added at sub-MIC concentrations (1/2 MIC, 1/4 MIC), and a growth control without Melittin was included. Subcultures were incubated at 37 °C with shaking at 240 rpm until they reached mid-log phase.

Bacterial pellets were collected by centrifugation at 4225× *g* for 1 h at 4 °C and stored at −80 °C until further analysis. β-lactamase activity was quantified using the chromogenic cephalosporin nitrocefin (β-lactamase Activity Assay Kit, Sigma-Aldrich, St. Louis, MO USA). Bacterial pellets were sonicated and centrifuged, and the supernatants were diluted 1:40 for kinetic assays. OD was measured at 490 nm in kinetic mode for 15 min at room temperature. Enzymatic activity was normalized to protein concentration, determined via the Bradford assay (BioRad, Hercules, CA, USA). The percentage of β-lactamase activity and its inhibition were calculated as follows:

$$\beta -lactamasas\;activity\;(\%)=\frac{\;\beta -lactamasa\;activity\;with\;Api<10\;\mathrm{kDa}\;}{Control\;\beta -lactamasa\;activity}\times 100$$

$$Inhibition\;\beta -lactamasas\;activity\;\left(\%\right)=100\%-Actividad\;\beta -lactamasas\;(\%)$$

Biological duplicates were performed for each strain and treatment, with three technical replicates analyzed per biological replicate.

### 4.6. Effect of Melittin on bla_CTX-M_ RNA Expression

#### 4.6.1. RNA Extraction

The impact of sub-MIC concentrations of Melittin on *bla*_CTX-M_ expression was assessed in *E. coli* strains producing CTX-M-type ESBLs. Early-log phase cells were cultured in CA-MHB containing Melittin (at 1/2 MIC or 1/4 MIC) at 37 °C until they reached mid-log phase. Total RNA was extracted using the GeneJET RNA Purification Spin Column Kit (Thermo Fisher Scientific, Waltham, MA, USA) following the manufacturer’s protocol, with an additional treatment using Turbo™ DNase (Invitrogen, Thermo Fisher Scientific, Carlsbad, CA, USA) to remove residual DNA. RNA concentrations were measured with a Qubit fluorometer (Invitrogen, Thermo Fisher Scientific).

#### 4.6.2. Quantitative Real-Time PCR (RT-qPCR)

Expression levels of the *bla*_CTX-M_ gene were quantified using the iTaq Universal SYBR^®^ Green One-Step Kit (BioRad, USA) following the manufacturer’s protocol. Specific primers targeting *bla*_CTX-M_ Group 1 [49] and the 16S rRNA gene [50] (internal control) were used. The PCR conditions included an initial cycle at 50 °C for 10 min, followed by 1 cycle at 95 °C for 1 min, and 40 amplification cycles at 95 °C for 10 s and 60 °C for 30 s. Relative gene expression changes were determined using the Livak 2^−ΔΔCt^ method [51]. Biological duplicates with two technical replicates each were included.

#### 4.6.3. Statistical Analysis

Data were analyzed using GraphPad Prism version 8 (GraphPad Software Inc., La Jolla, CA, USA). The Friedman test was applied to evaluate differences in ESBL enzymatic activity and gene transcription profiles between control and treatment groups. Linear regression analysis was used to compare the slopes of bacterial growth curves. A *p*-value < 0.05 was considered statistically significant.

## Figures and Tables

**Table 1:** Minimum inhibitory concentration (MIC) and sub-inhibitory doses of Melittin against CTX-M-type ESBL-producing *E. coli*.

Cepa	MIC µg/mL	1/2 MIC µg/mL	1/4 MIC µg/mL
5B	60	30	15
11B	60	30	15
12B	50	25	12.5
14B	80	40	20
16B	60	30	15

**Table 2:** Hydrolytic activity of CTX-M-type ESBL-producing *E. coli* following exposure to Melittin based on each strain’s MIC.

Hydrolytic Activity of β-Lactamases mU/mg Protein ^a^			
Strain	Control	1/4MIC	1/2MIC ^b^
5B	4327	4589 (−6.1%)	3292 (23.9%)
11B	2561	2745 (−7.2%)	1684 (34.2%)
12B	3322	3671 (−10.5%)	2156 (35.1%)
14B	897	607 (32.3%)	294 (67.2%)
16B	1438	977 (32.1%)	872 (39.4%)

^a^ The specific activity of β-lactamases is presented as nmoles of nitrocefin hydrolyzed per minute per milligram of protein. The percentage of β-lactamase enzyme inhibition relative to the control is shown in parentheses. ^b^ *p* < 0.05.

## Data Availability

The information used in our study is available upon request from the corresponding author. The dataset is not available to the public due to the need to protect information.
